# The Effects of Interstitial Lung Diseases on Alveolar Extracellular Vesicles Profile: A Multicenter Study

**DOI:** 10.3390/ijms24044071

**Published:** 2023-02-17

**Authors:** Miriana d’Alessandro, Sara Gangi, Piera Soccio, Elisabet Cantó, Rubén Osuna-Gómez, Laura Bergantini, Paolo Cameli, Gaia Fabbri, Sara Croce, Giulia Scioscia, Giusy Montuori, Matteo Fanetti, Giorgia Moriondo, Fabrizio Mezzasalma, Diego Castillo, Donato Lacedonia, Silvia Vidal, Elena Bargagli

**Affiliations:** 1 Respiratory Diseases and Lung Transplantation Unit, Department of Medical and Surgical Sciences & Neuro-Sciences, University of Siena, 53100 Siena, Italy; 2 Department of Medical and Surgical Sciences, University of Foggia, 71122 Foggia, Italy; 3 Institute of Respiratory Diseases, Policlinico Riuniti of Foggia, 71122 Foggia, Italy; 4 Inflammatory Diseases, Biomedical Research Institute Sant Pau (IIB Sant Pau), 08041 Barcelona, Spain; 5 Diagnostic and Interventional Bronchoscopy Unit, Cardio-Thoracic and Vascular Department, University Hospital of Siena (Azienda Ospedaliera Universitaria Senese—AOUS), 53100 Siena, Italy; 6 Respiratory Department, Hospital de la Santa Creu i Sant Pau, Sant Pau Biomedical Research Institute (IIB-Sant Pau), 08041 Barcelona, Spain

**Keywords:** extracellular vesicles, interstitial lung diseases, bronchoalveolar lavage, idiopathic pulmonary fibrosis, diagnosis

## Abstract

Diagnosis of interstitial lung diseases (ILD) is difficult to perform. Extracellular vesicles (EVs) facilitate cell-to-cell communication, and they are released by a variety of cells. Our goal aimed to investigate EV markers in bronchoalveolar lavage (BAL) from idiopathic pulmonary fibrosis (IPF), sarcoidosis and hypersensitivity pneumonitis (HP) cohorts. ILD patients followed at Siena, Barcelona and Foggia University Hospitals were enrolled. BAL supernatants were used to isolate the EVs. They were characterized by flow cytometry assay through MACSPlex Exsome KIT. The majority of alveolar EV markers were related to the fibrotic damage. CD56, CD105, CD142, CD31 and CD49e were exclusively expressed by alveolar samples from IPF patients, while HP showed only CD86 and CD24. Some EV markers were common between HP and sarcoidosis (CD11c, CD1c, CD209, CD4, CD40, CD44, CD8). Principal component analysis distinguished the three groups based on EV markers with total variance of 60.08%. This study has demonstrated the validity of the flow cytometric method to phenotype and characterize EV surface markers in BAL samples. The two granulomatous diseases, sarcoidosis and HP, cohorts shared alveolar EV markers not revealed in IPF patients. Our findings demonstrated the viability of the alveolar compartment allowing identification of lung-specific markers for IPF and HP.

## 1. Introduction

Among the non-neoplastic lung diseases, interstitial lung diseases (ILD) represent a wide and heterogeneous group of more than 200 distinct clinical entities, characterized by the specific involvement of lung interstitium that may lead to pulmonary fibrosis [1].

In the case of ILD, identifying the disease in a precise and timely manner can often be challenging for many reasons: pathophysiological disease mechanisms are inherently complex, classification systems and definitions are constantly evolving, overlapping conditions are present within the lung and multiple pathological entities may coexist in the same lung [2]. As a result of the latest classification systems, an open dialogue has been developed regarding ILD and a common language has been developed for communicating and interpreting research findings [1]. A multidisciplinary approach is currently the gold standard for diagnosing complex ILD cases, which includes the assessment of clinicians, radiologists, and pathologists [3,4]. Interobserver disagreement regarding diagnoses remains significant, however, as more data are included in the assessment, this disagreement improves [5]. When combined with clinical data and high-resolution computed tomography (HRCT), bronchoscopy with bronchoalveolar lavage (BAL) and subsequent differential cell counts, microbiological tests and cytopathology are generally considered valuable for diagnosing ILD [6,7]. Although BAL is seldom used as a self-standing diagnostic test, it can still be used to confirm or exclude certain diagnoses based on its characteristics regarding the main immune cell populations and the soluble factors contained in the BAL fluid [6].

Extracellular vesicles (EVs) facilitate cell-to-cell communication, which plays a vital role in nearly all physiological and metabolic activities [8]. As a general term, EVs refer to particles released by cells that are not contained within a lipid bilayer and cannot reproduce. Cell membranes secrete EVs into circulation and body fluids, and they are abundant in respiratory fluid samples [9], including BAL. In addition to their cytoplasmatic components, EVs possess surface proteins that allow them to adhere to circulating or distant cells and fuse them together. In order to function as intercellular messengers, they are able to transfer signalling molecules [10].

The endothelium of the lung contributes significantly to EV circulation due to its high vascular density [11]. In addition, alveolar macrophages, fibroblasts, epithelial cells, granulocytes and lymphocytes may also produce EVs [11]. Using intracellular communicators provides valuable insight into the state of health and disease in cells. They can also serve as biomarkers for lung diseases, including fibrotic ones [8]. 

Our research group has recently conducted EV analyses in peripheral blood of patients with idiopathic pulmonary fibrosis (IPF) using flow cytometry and showing an altered exosome profile [9]. These findings emphasized the need to conduct comparative studies in different anatomical compartments, including the profile of exosomes at the site of lung injury.

The present study aimed to compare 37 exosomal surface markers through flow cytometry in BAL from patients affected by IPF, sarcoidosis and hypersensitivity pneumonitis (HP), enrolled at Siena Referral Centre for rare lung diseases. To corroborate these findings, a validation cohort was enrolled from two referral centers for ILDs (Barcelona and Foggia).

## 2. Results

### 2.1. Patients

Demographic, clinical and immunological data of study and validation cohorts are reported in Table 1. All patients were enrolled at the moment of diagnosis and were not taking any pharmacological treatment for lung disease at the moment of inclusion. All cohorts showed a prevalence of males in IPF than other ILD (*p* = 0.0312), while sarcoidosis patients were younger (*p* = 0.0215) and with a female predominance than IPF and HP (*p* = 0.0121). According to respiratory functional assessment at baseline, we observed a moderate reduction of DLCO percentages associated with mild restrictive impairment of lung volumes in IPF patients compared with HP and sarcoidosis (*p* = 0.0399). 

### 2.2. EVs Surface Markers 

From comparative analysis, the expression of EV surface markers was similar in the cohort and validation cohort (*p* = 0.860). CD133/1, SSEA, CD14 and CD2 were differently expressed in BAL samples from the Siena and Foggia cohorts than the Barcelona cohort (*p* = 0.0455). The expression of CD9, CD63 and CD81 were similar in both cohorts (*p* = 0.905). 

MANOVA analysis between IPF, HP and sarcoidosis associated lambda (0.174) with a *p*-value (*p* < 0.0001), indicating a risk of erroneously rejecting the null hypothesis lower than 0.01%.

**Figure 1:**
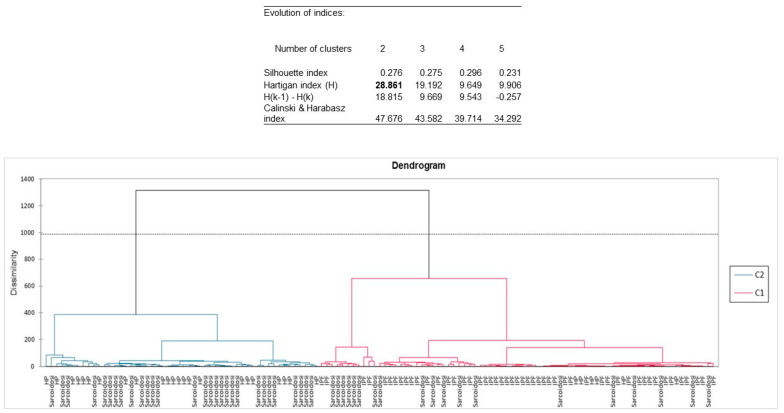
Dendogram plot from agglomerative hierarchical clustering (AHC) analysis clustered the three groups of diagnoses (IPF, HP, sarcoidosis) based on dissimilarities between the median APC (allophycocyanin) values of 37 EV surface markers. Hartigan index defined 2 as the appropriate number of clusters because of the greater difference (the value displayed in bold). The first cluster (displayed in blue, mainly formed by granulomatous disease: HP and sarcoidosis) is more homogeneous than the second one (it is flatter on the dendrogram, mainly formed by IPF patients).

Assessing AHC analysis (Figure 1), the Hartigan index defined 2 as the appropriate number of clusters because of the greater difference (the value displayed in bold). The first cluster (displayed in blue, mainly formed by granulomatous disease: HP and sarcoidosis) is more homogeneous than the second one (it is flatter on the dendrogram, mainly formed by IPF patients). 

**Figure 2:**
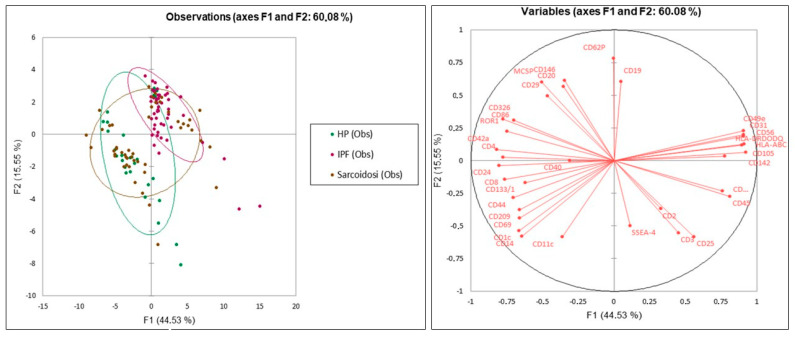
Unsupervised principal component analysis (PCA) to distinguish the three groups (IPF, HP and sarcoidosis) on the basis of 37 surface exosome markers with total variance of 60.08%.

PCA plots (Figure 2) were performed to distinguish the three groups (IPF, HP and sarcoidosis). The analysis showed that the three groups were separated on the basis of 37 surface exosome markers. The first and second components explained 44.53% and 15.55% of the total variance.

**Figure 3:**
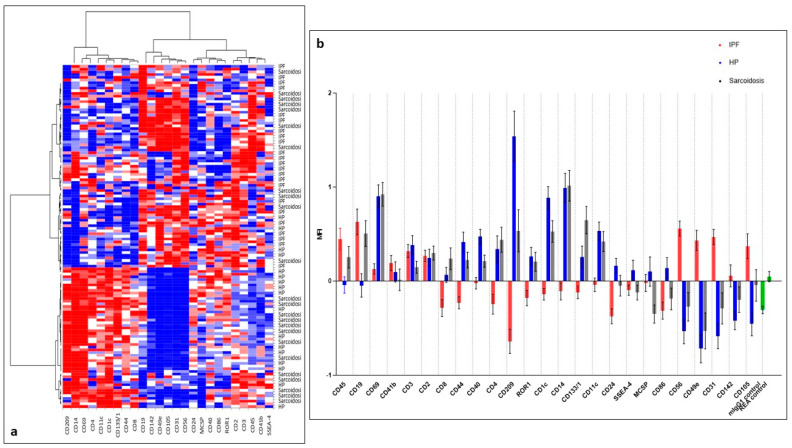
Heat map analysis (**a**) showed that the significant EV surface markers may be expressed in clusters of IPF, HP and sarcoidosis; blue to red through white (Blue: −1 / White: 0 / Red: 1). The median APC (allophycocyanin) values of each significant different surface marker in IPF, HP and sarcoidosis were reported in (**b**). Abbreviations: CD-, cluster of differentiation; IPF, idiopathic pulmonary fibrosis; HP, hypersensitivity pneumonitis.

Heat map analysis (Figure 3a) showed that the significant EV surface markers may be expressed in clusters of IPF, HP and sarcoidosis. The median APC values of each significant different surface marker in IPF, HP and sarcoidosis were reported in Figure 3b.

**Figure 4:**
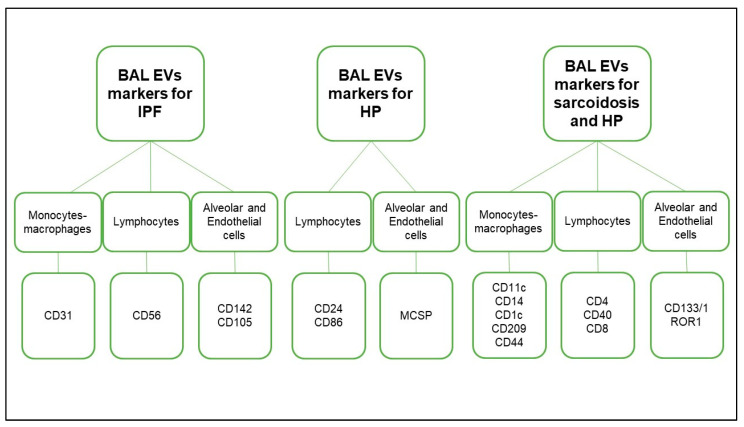
The main significant EV markers divided according to the diseases (IPF, HP and sarcoidosis) and their sources: monocytes/macrophages, lymphocytes, alveolar and endothelial cells. Abbreviations: EVs, extracellular vesicles; IPF, idiopathic pulmonary fibrosis; HP, hypersensitivity pneumonitis; CD-, cluster of differentiation.

Figure 4 shows the main markers expressed exclusively in IPF (CD105, CD142, CD31, CD49e and CD56), only in HP (CD86, MCSPand CD24) as well as those markers that are common between HP and sarcoidosis (CD11c, CD133/1, CD14, CD1c, ROR1, CD209, CD4, CD40, CD44, CD8) and in both BAL samples of IPF and sarcoidosis (CD19 and CD45) BAL samples.

## 3. Discussion

**Figure 5:**
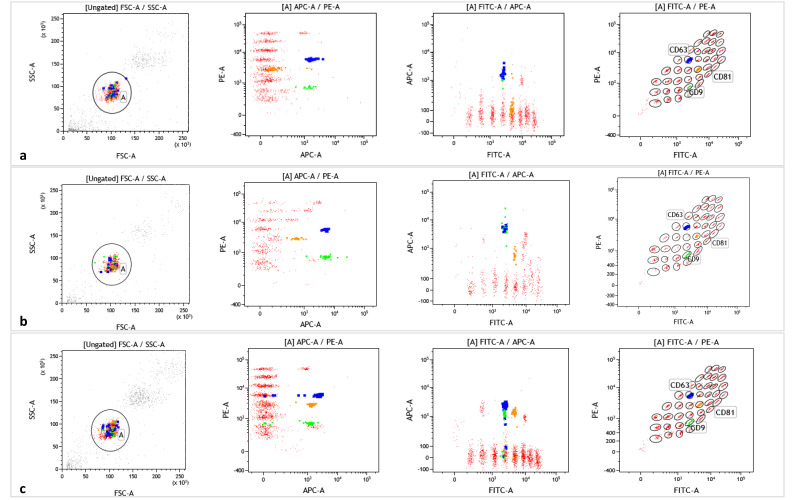
Gating strategy of exosome surface markers from the Siena cohort (**a**), Foggia cohort (**b**) and Barcelona cohort (**c**) analyzed using Kaluza Software 2.1 (Beckman Coulter, Cassina De’ Pecchi–Milano, Italy).

In the present multicenter study, 37 EV surface markers were detected in the alveolar compartment of ILD patients at diagnosis for the first time. IPF, HP and sarcoidosis patients were sampled for BALs and EVs were phenotyped by flow cytometric analysis. The majority of markers identified on EV surfaces were related to the fibrotic damage on alveolar epithelial cells I and II as well as endothelial cells and fibroblasts, as reported in Figure 5. The others concerned lymphocytes and monocytes/macrophages, probably due to the retrieval at the site of lung injury (Figure 5). EVs from IPF patients expressed CD56 primarily in the alveolar compartment, even if CD69 expression was lower in IPF than HP and sarcoidosis BAL samples. The former is an adhesion molecule expressed on NK cells that promotes their cytotoxicity [12] and the latter is an activation marker [13]. Studies have demonstrated that peripheral blood NK cells were higher in IPF patients than those from other ILD and that NK cell activity may be detrimental in terms of disease progression [14,15], though they did not find differences in BAL samples. In our previous study, we identified a higher CD69 expression on peripheral blood from IPF patients [9]. Taken together, such findings suggest that peripheral cellular environments may have a relation to pulmonary resident cells, thus emphasizing the possibility of systemic involvement in IPF natural course. 

IPF etiopathogenesis is still unknown. A dysfunctional epithelium can be ascribed to genetics and epigenetics [16]. As a result of this altered epithelium, recurrent micro-lesions resulting from environmental exposures such as cigarette smoke, dust inhalation, infections and gastroesophageal reflux are more likely to occur [17,18]. Additionally, the epithelium appears to lose its normal ability to regenerate following repeated trauma over time, which appears to contribute to the progression of IPF [19,20]. Alveolar and capillary walls are altered and destroyed. Activating the coagulation cascade and resulting abnormal vascular remodeling are part of the attempt to repair damaged capillaries by leaking proteins such as fibrin and fibronectin into interstitial and alveolar spaces [21]. The fibrotic process is thus still influenced by neoangiogenesis [22]. The repeated stimulation of type II alveolar epithelial cells (AEC2) has been shown to primarily damage these cells [23]. This alters the relationship between epithelial cells and fibroblasts, increases the extracellular matrix, remodels the interstitium and produces fibroblast foci [24,25]. The expression of CD142 (known as tissue factor or coagulation factor III/thromboplastin, mainly expressed on AECII) [26] in IPF BAL samples, rather than HP or sarcoidosis samples, supported the involvement of coagulation cascade in the fibrotic process [27]. In addition, only IPF BAL samples had EVs expressing higher CD49e (a receptor of fibronectin and fibrinogen that is primarily expressed by endothelial cells [28]) and CD105 (a TGF-beta receptor involved in neoangiogenesis [29]) than HP and sarcoidosis EV BAL samples. Higher expression of CD41b [30], a receptor for fibronectin, fibrinogen, plasminogen, prothrombin, thrombospondin and vitronectin, was reported in IPF patients compared to sarcoidosis and HP BAL samples. According to the present study, the EV markers identified in BAL samples from IPF patients were not found in previous studies with peripheral samples indicating that these markers are lung specific. Furthermore, CD31 [31], an adhesion molecule involved in transendothelial migration of neutrophils, was expressed in EV BAL samples from IPF patients. This finding is surely interesting in terms of prognosis and disease progression, since an increased BAL neutrophil percentage at diagnosis of IPF was reported as an independent predictor of time to death or transplant [32,33,34,35]. 

It has been hypothesized that HP and IPF have distinct lung fibrosis phenotypes, one being a post-inflammation-induced fibrosis, whereas the second is more related to tissue remodeling and repair [36,37].

The expression of a number of cell- and inflammation-related markers in our study supports the hypothesis that inflammation plays a significant role in the pathogenesis of HP and sarcoidosis.

In HP, T-lymphocyte markers, CD3, were higher than in sarcoidosis and IPF. In BAL samples from sarcoidosis patients, however, the highest expression of CD4 and CD8 was associated with alveolar lymphocytosis, indicating that the disease was active at the time of diagnosis. Sarcoidosis patients have demonstrated increased inflammatory responses in circulating blood monocytes [38]. It is likely that there is an interaction with both adaptive immunity and innate immunity in sarcoidosis, as evidenced by the presence of T-lymphocytes and macrophages derived from monocytes [39]. Adhesion molecules for monocytes and macrophages’ marker, CD11c [40] and CD1c [41], were more prominent in our sarcoidosis cohort compared to alveolar samples from HP but absent in IPF samples. Inflammatory responses are mediated by such markers, and an important research focus will be the identification of the drivers of non-resolving and progressive sarcoidosis, which are accompanied by considerable morbidity and loss of quality of life [42].

Alveolar samples from HP patients showed the highest expression of several B-lymphocytes markers, including CD40 (extracellular positivity in lymphoid tissues [43]) and CD24 (module B-cell activation [44]). 

There are both T and B cells in the lung parenchyma, and their organization into B lymphoid-rich tertiary lymphoid tissues is a hallmark of HP that is closely related to the level of airway inflammation [45]. Stimulated B cells (plasma cells) produce IgG antibodies, which initiates the complement cascade and further stimulates macrophages, as confirmed by our study that reports the expression of CD44 (adhesion molecule expressed by granulocytes and alveolar macrophages [46]) and CD209 (marker for macrophages M2 [47]). It is widely recognized that the formation of immune complexes in HP is related to the complement system, macrophage activation and the subsequent promotion of airway neutrophilic inflammation, and that detecting circulating agent-specific antibodies remains an important aspect of diagnosis and treatment [48].

It is important to emphasize that even though our study provides a relevant contribution by highlighting the expression markers of EVs in BAL samples, it has some limitations. First, we did not include other fibrotic ILD in our study that may show a similar clinical course to IPF or HP. Second, it is important to validate our findings in other ILD cohorts from different geographical areas. In addition, the analysis of EV surface markers in different biological samples will be of interest. 

## 4. Materials and Methods

### 4.1. Study Cohort

A total of 83 (mean age ± standard deviation, 65 ± 23.4) patients from the rare lung diseases referral center of the University of Siena were consecutively enrolled in the study. A multidisciplinary discussion confirmed the diagnosis of IPF and fibrotic HP in seventeen (mean age ± standard deviation, 64.7 ± 23.8) and twenty-four (mean age ± standard deviation, 78.1 ± 25.2) patients, respectively, according to the American Thoracic Society/European Respiratory Society (ATS/ERS) guidelines [1,3]. Forty-two patients (mean age ± standard deviation, 52.2 ± 21.2) were diagnosed as having sarcoidosis according to international criteria based on clinical signs, chest radiography findings and non-caseating granulomas in lymph nodes and/or endobronchial biopsy specimens and were confirmed through multidisciplinary evaluation as well [4,49]. Infectious and malignant diseases were excluded. Demographic and clinical data, including comorbidities, family history, lung function parameters and radiological features were obtained from the medical records and entered into an electronic database for statistical analysis. 

This study was approved by the regional ethical review board of Siena (C.E.A.V.S.E. Markerlung 17431) and complied with the Helsinki Declaration. All patients provided written informed consent prior to participating in the study. Healthy donors were not enrolled for ethical reasons.

### 4.2. Validation Cohort

Twenty-one patients (mean age ± standard deviation, 61 ± 18.3) referred to ILD clinic at Hospital de la Santa Creu i Sant Pau (Barcelona) were retrospectively enrolled. Eleven patients were affected by IPF, 5 by sarcoidosis and 5 by HP. 

Forty-four patients (mean age ± standard deviation, 62 ± 19.5) followed at ILD referral center of Foggia University Hospital were consecutively and prospectively enrolled. Thirty-three patients had diagnoses of IPF, 5 of sarcoidosis and 6 of HP. 

All diagnoses were made according to ATS/ERS international guidelines based on multidisciplinary discussion.

### 4.3. Pulmonary Function Test Parameters

Pulmonary function tests (PFT) were performed in all patients for diagnostic purposes. Forced expiratory volume in the first second (FEV1), forced vital capacity (FVC) and diffusing capacity of the lung for carbon monoxide (DLCO) were recorded. They were performed according to ATS/ERS standards [50] using a Jaeger body plethysmograph with correction for temperature and barometric pressure. 

### 4.4. Bronchoscopy

Patients were treated with fentanyl 100 mcg and midazolam (3–5 mg) i.v. 15–30 min prior to bronchoscopy. Lidocaine was administered to the larynx and bronchi for topical anesthesia. A Pentax bronchoscope EB15-J10 (Pentax Medical Company, PENTAX Europe GmbH, Hamburg, Germany) was inserted through the mouth to avoid blood contamination. Physiological saline with 3 × 50 mL solution was instilled in the middle or lingual lobe. Ten to twenty mL of a pooled BAL sample were collected, as recommended by international BAL Task Force Group guidelines for BAL cellular analysis [51]. BAL samples were processed as follows: BAL was filtered through sterile gauze and cell count was determined by cytocentrifuge smear (600 rpm for 5 min) with a Thermo Shandon Cytospin 3 (Marshall Scientific, Hampton, NH, USA), and stained with Diff quik stain kit (DiaPath, Martinengo, BG, Italy); a total of 500 cells was counted distinguishing macrophage, lymphocyte and neutrophil percentages. Cell viability was determined by Trypan blue exclusion in a Burker Chamber. BAL samples containing 5% or less of ciliated columnar epithelial cells were considered suitable.

### 4.5. EVs Isolation and Characterization

BAL samples were processed as follow: the samples were centrifuged at 1500× *g* for 10 min and then the supernatant was stored at −80 °C. According to the manufacturer’s instruction (MACSPlex Exosome kit, Miltenyi Biotec, Bergisch Gladbach, Germany) [52], 120 μL of supernatant was centrifuged at 2000× *g* for 30 min at room temperature (RT), then 110 μL of each sample was aspirated and centrifuged at 10,000× *g* for 45 min at RT. EVs were characterized by MACSPlex Exosome Capture (Miltenyi Biotec, Bergisch Gladbach, Germany) beads and incubated overnight using an orbital shaker (450 rpm). Then, 500 μL of MACSPlex Buffer was added in each tube and centrifuged at RT at 300× *g* for 5 min. Accordingly, each sample was then incubated with CD9, CD63 and CD81 for 1 h at RT protected from light on the orbital shaker. The washing step was repeated and EVs from Siena and Foggia University Hospitals were characterized by FACSLyric (BD Biosciences) and analyzed by Kaluza software 2.1 (Beckman Coulter, Cassina De’ Pecchi–Milano, Italy), while EVs isolated at Barcelona Hospital were characterized using a MACSQuant Analyzer 16 Flow Cytometer (Miltenyi Biotec, Bergisch Gladbach, Germany). According to literature data, the results from the two flow cytometers are accurate and comparable [53,54]. The gating strategy obtained by Kaluza software 2.1 (Beckman Coulter, Cassina De’ Pecchi–Milano, Italy) to detect exosome surface markers is reported in Figure 5.

### 4.6. Statistical Analysis

The results were expressed as means and standard deviations (SD). One-way ANOVA non-parametric test (Kruskal–Wallis test) and Dunn test were performed for multiple comparisons. The chi-squared test was used for categorical variables. 

MANOVA analysis was performed to determine any significant effects of IPF, HP and sarcoidosis diagnoses (explanatory variables) considered in interaction or otherwise with 37 EV surface markers (dependent variables). Wilks’ lambda tests were performed, the lower the lambda associated with explanatory variables, the more important the effect of these variables is on the dependent variables’ combination. 

Agglomerative hierarchical clustering (AHC) was performed to cluster the three groups of diagnoses based on dissimilarities between the median APC (allophycocyanin) values of 37 EV surface markers. The dendrogram plot was assessed showing the progressive grouping of the data. 

Unsupervised principal component analysis (PCA) and heat map analysis were applied to surface expression markers in an explorative approach to identify trends in the data by 2D representation of the multi-dimensional data set.

A *p* value less than 0.05 was considered statistically significant. Statistical analysis was performed using GraphPad Prism 9.4 and XLSTAT2021 software.

## 5. Conclusions

The findings of our study have demonstrated the validity of the flow cytometric method to phenotype and characterize EV surface markers in BAL samples. In addition, the viability of the alveolar compartment, as a result of the BAL, was demonstrated, which allowed identifying lung-specific markers for IPF and HP. A further outcome of this study was corroboration of the hypotheses regarding the different fibrotic phenotypes in HP and IPF, where the first is related to cell-mediated inflammation-induced lung disease, while the latter is more often attributed to tissue repair and remodeling. The EV markers identified in BAL samples from IPF patients were not revealed in the HP cohort, most of whom were shared with sarcoidosis as two granulomatous ILD.

## Figures and Tables

**Table 1:** Demographic and clinical data including age, gender and smoking habit as well as BAL cellular patterns and PFT parameters in study and validation cohorts. All parameters were expressed as mean ± standard deviation. ^1^ Prevalence of younger in sarcoidosis than HP and IPF, *p* = 0.0215; ^2^ prevalence of female in sarcoidosis than IPF and HP, *p* = 0.121; ^3^ reduction of DLCO percentages in IPF than sarcoidosis and HP, *p* = 0.0399. Abbreviations: IPF, idiopathic pulmonary fibrosis; HP, hypersensitivity pneumonitis; BAL, bronchoalveolar lavage; FVC, forced vital capacity; FEV1, forced expired volume in 1 s; DLco, diffuse lung monoxide carbon; L, liters.

Parameters	Study Cohort			Validation Cohort		
	IPF (*n* = 17)	HP (*n* = 24)	Sarcoidosis (*n* = 42)	IPF (*n* = 44)	HP (*n* = 11)	Sarcoidosis (*n* = 10)
Age (years)	64.7 ± 23.8	68.23 ± 11.9	52.2 ± 21.2 ^1^	62.6 ± 19.8	67.11 ± 9.2	50.2 ± 20.5 ^1^
Gender (male/female)	13/4	14/10	16/36 ^2^	31/13	6/5	3/7 ^2^
Smoking habit (never/former)	6/11	6/18	15/27	15/28	4/7	4/6
BAL cellular pattern						
Macrophages	71.8 ± 23.4	76.5 ± 16.0	67.0 ± 19.5	59.7 ± 22.4	47.6 ± 18.4	40.0 ± 14.1
Lymphocytes	13.4 ± 16.6	14.5 ± 14.8	21.1 ± 14.8	10.1 ± 5.07	19.0 ± 5.48	33.0 ± 14.7
Neutrophils	11.4 ± 11.4	5.46 ± 5.03	11.2 ± 20.9	19.2 ± 19.1	28.0 ± 20.4	10.8 ± 5.68
Pulmonary function test parameters						
FVC %	81.9 ± 27.5	69.1 ± 22.2	99.3 ± 19.5	79.6 ± 10.4	63.6 ± 13.2	93.8 ± 8.73
FVC L	2.44 ± 0.573	2.37 ± 0.992	3.51 ± 1.16	3.27 ± 0.598	2.47 ± 0.951	4.59 ± 0.667
FEV1 %	84.9 ± 22.0	72.0 ± 21.2	95.4 ± 20.3	81.6 ± 12.0	60.8 ± 10.6	90.0 ± 8.29
FEV1 L	2.04 ± 0.475	1.98 ± 0.757	5.83 ± 16.3	2.48 ± 0.570	1.76 ± 0.715	3.39 ± 0.472
DLco %	51.4 ± 21.2 ^3^	58.4 ± 28.5	78.2 ± 20.9	52.6 ± 19.8 ^3^	59.2 ± 32.3	75.7 ± 20.5
DLco L	3.82 ± 1.30	5.14 ± 21.2	7.62 ± 1.92	4.4 ± 4.93	6.7 ± 8.90	10.7 ± 6.93

## Data Availability

The data presented in this study are available on request from the corresponding author.

## Abbreviations

EVs, extracellular vesicles; BAL, bronchoalveolar lavage; ILD, interstitial lung disease; IPF, idiopathic pulmonary fibrosis; HP, hypersensitivity pneumonitis; HRCT, high-resolution computed tomography; IQR, interquartile range; *p* value, probability value; AHC, agglomerative hierarchical clustering; PCA, principal component analysis; CD-, cluster of differentiation; NK, natural killer; AEC-, alveolar epithelial cells.
